# Spontaneous Ca^2+^ Influx in *Drosophila* Pupal Neurons Is Modulated by IP_3_-Receptor Function and Influences Maturation of the Flight Circuit

**DOI:** 10.3389/fnmol.2017.00111

**Published:** 2017-04-20

**Authors:** Sumita Chakraborty, Gaiti Hasan

**Affiliations:** National Centre for Biological Sciences, Tata Institute of Fundamental ResearchBangalore, India

**Keywords:** SOCE, STIM, Orai, VGCC, Trp

## Abstract

Inositol 1,4,5-trisphosphate receptors (IP_3_R) are Ca^2+^ channels on the neuronal endoplasmic reticulum (ER) membrane. They are gated by IP_3_, produced upon external stimulation and activation of G protein-coupled receptors on the plasma membrane (PM). IP_3_-mediated Ca^2+^ release, and the resulting depletion of the ER store, triggers entry of extracellular Ca^2+^ by store-operated Ca^2+^ entry (SOCE). Mutations in IP_3_R attenuate SOCE. Compromised IP_3_R function and SOCE during pupal development of *Drosophila* leads to flight deficits and mimics suppression of neuronal activity during pupal or adult development. To understand the effect of compromised IP_3_R function on pupal neuronal calcium signaling, we examined the effects of mutations in the IP_3_R gene (*itpr*) on Ca^2+^ signals in cultured neurons derived from *Drosophila* pupae. We observed increased spontaneous Ca^2+^ influx across the PM of isolated pupal neurons with mutant IP_3_R and also a loss of SOCE. Both spontaneous Ca^2+^ influx and reduced SOCE were reversed by over-expression of *dOrai* and *dSTIM*, which encode the SOCE Ca^2+^ channel and the ER Ca^2+^-sensor that regulates it, respectively. Expression of voltage-gated Ca^2+^ channels (*cac, Ca-α1D* and *Ca-αT*) was significantly reduced in *itpr* mutant neurons. However, expression of *trp* mRNAs and transient receptor potential (TRP) protein were increased, suggesting that TRP channels might contribute to the increased spontaneous Ca^2+^ influx in neurons with mutant IP_3_R. Thus, IP_3_R/SOCE modulates spontaneous Ca^2+^ influx and expression of PM Ca^2+^ channels in *Drosophila* pupal neurons. Spontaneous Ca^2+^ influx compensates for the loss of SOCE in *Drosophila* *itpr* mutant neurons.

## Introduction

Inositol 1, 4, 5-trisphosphate receptors (IP_3_R) are Ca^2+^ channels on the neuronal endoplasmic reticulum (ER) membrane. IP_3_Rs are gated by the second messenger IP_3_, which is produced upon external stimulation and activation of G protein-coupled receptors (GPCRs) on the plasma membrane (PM). IP_3_-mediated Ca^2+^ release, and the resulting depletion of the ER store, triggers entry of extracellular Ca^2+^ by store-operated Ca^2+^ entry (SOCE). *Drosophila* mutants for the IP_3_R are flightless (Banerjee et al., 2004). Pan-neuronal knockdown of *itpr* (the gene for IP_3_R) and of genes encoding other calcium signaling molecules, such as GPCRs and the SOCE molecules, *dSTIM* and *dOrai* also result in flight deficits (Venkiteswaran and Hasan, 2009; Agrawal et al., 2010, 2013). These and other studies have shown that maturation of the flight circuit during pupal development requires intracellular calcium signaling through the IP_3_R followed by SOCE. This signaling is initiated by GPCRs and affects the transcriptional profile of developing flight circuit neurons (Agrawal et al., 2010, 2013; Pathak et al., 2015). Flight deficits in *Drosophila* with reduced intracellular calcium signaling during pupal stages, also correlate with reduced levels of Tyrosine Hydroxylase in dopaminergic neurons (Pathak et al., 2015), suggesting that calcium signaling through the IP_3_R in pupal neurons modulate neurotransmitter levels in adult *Drosophila*. In vertebrate neurons, neurotransmitter specification is modulated by calcium signaling through voltage-gated Ca^2+^ channels (VGCCs) and can be decoded by the frequency and amplitude of spontaneous Ca^2+^ transients (Spitzer et al., 2005; Dulcis et al., 2013); lower for excitatory neurotransmitters and higher for inhibitory neurotransmitters. Spontaneous Ca^2+^ signals (transients and sustained) in *Drosophila* pupal neurons are also mediated through VGCCs (Jiang et al., 2005; Iniguez et al., 2013). The TRPC class of PM Ca^2+^ channels function as polymodal cellular sensors and mediate changes in membrane voltage and intracellular calcium signals. In the developing *Xenopus* spinal cord TRPC channels are responsible for Ca^2+^ spike activity (Belgacem and Borodinsky, 2011). However a role for TRPC in generating spontaneous Ca^2+^ signals in *Drosophila* neurons is unknown.

Spontaneous Ca^2+^ oscillations in *Drosophila* occur in intact as well as in isolated brains, indicating that these signals are independent of sensory inputs (Rosay et al., 2001). In mushroom body Kenyon cells, frequency of spontaneous Ca^2+^ transients in isolated pupal neurons is similar to Ca^2+^ transients *in vivo* (Jiang et al., 2005). Here we have investigated the nature of spontaneous Ca^2+^ signals in cultured *Drosophila* pupal neurons where IP_3_/SOCE mediated intracellular Ca^2+^ signaling is disrupted. We show that pupal neurons from *itpr* mutants exhibit aberrantly high spontaneous Ca^2+^ influx and reduced SOCE. We propose that in *itpr* mutant neurons, higher spontaneous Ca^2+^ influx functions as a compensatory mechanism for decreased SOCE and helps maintain intracellular Ca^2+^ homeostasis. A possible source of the compensatory spontaneous Ca^2+^ influx appears to be the TRP channel.

## Materials and Methods

### *Drosophila* Strains

Single point mutants in the *itpr* gene were generated in an EMS (ethyl methane sulfonate) screen; detailed molecular information on these alleles has been published (Joshi et al., 2004; Srikanth et al., 2004). The *UAS* transgenic strains used have been published and the appropriate references are included in the results. *Elav^C155^GAL4* (pan neuronal) was obtained from Bloomington Stock Center, Indiana University, Bloomington, IN, USA. *Canton S* (*CS*), in the background of which all mutant and transgenics were back-crossed, was used as the wild-type control. RNAi lines for *itpr* (1063) was obtained from National Institute of Genetics, Japan, and for *dSTIM* (47073), *cac* (104186), *Ca-β* (102188), *Ca-α1D* (51491) and *Ca-α1T* (48008, 31961) were obtained from Vienna Drosophila Resource Centre, Austria. Fly strains used in this study were generated by standard genetic methods using individual mutant and transgenic fly lines described above.

### Primary Neuronal Culture

The protocol for pupal neuronal culture was adapted from Sicaeros and O’Dowd (2007). The brain and ventral ganglion complexes were dissected from different pupal stages of the appropriate genotypes. Dissected brain tissues were incubated for 15 min at RT with 50 U/ml papain activated by 1.32 mM cysteine in dissecting saline (5.4 mM KCl/137 mM NaCl/0.22 mM KH_2_PO_4_/0.17 mM NaH_2_PO_4_/43.8 mM sucrose/33.3 mM glucose/9.9 mM HEPES, pH 7.3 with NaOH). The brain tissue was dissociated with gentle pipetting. The lysate containing a mixture of tissue clumps and single cells was spun down, re-suspended and plated onto poly lysine-coated glass coverslip mounted to the bottom of a petri dish. Cells were resuspended and cultured in DMEM/F12-1065 (Life Technologies, Carlsbad, CA, USA), containing Glutamax-I, 2.438 sodium bicarbonate and sodium pyruvate, supplemented with 50 U/ml penicillin (Life Technologies, Carlsbad, CA, USA), 50 μg/ml streptomycin (Life Technologies, Carlsbad, CA, USA), and 10 μg/ml amphotericin B (Life Technologies, Carlsbad, CA, USA), 1 mg/ml sodium bicarbonate, 20 mM HEPES, 100 μM putrescine, 20 ng/ml progesterone, 50 μg/ml insulin, 1 μg/ml 20-hydroxyecdysone. The cells were incubated at 25°C in a humidified incubator with 5% CO_2_ for 14–16 h. All chemicals for cell culture were obtained from Sigma-Aldrich (St. Louis, MO, USA) unless otherwise stated.

### Calcium Imaging

For measurement of spontaneous calcium influx and SOCE cells were incubated with 2.5 μM Fluo-4 AM and 0.02% Pluronic F-127 in M1 media for 30 min at room temperature in the dark. Cells were washed twice with M1 before and after dye incubation and finally covered with M1 (30 mM HEPES/150 mM NaCl/5 mM KCl/2 mM MgCl_2_/35 mM sucrose, pH 7.2 with NaOH) with (1 mM CaCl_2_) or without (2 mM EGTA) Ca^2+^. Data were acquired using 488 nm excitation and 520 nm emission filter sets at 15 s intervals. An Olympus IX81-ZDC2 inverted wide field microscope with epifluorescence and Z-drift compensation and a 60×/1.35 NA (oil) objective lens, was used for calcium imaging. Excitation of fluorescent Ca^2+^ indicator dyes was performed using specific wavelength illuminations from a halogen arc lamp with TILL Polychrome 5000 monochromator (TILL Photonics, Graefelfing, Germany) for variable bandwidth and intensity. Emitted light was detected through band pass filter sets (Chroma, Brattleboro, VT, USA). Image acquisition was performed using the Andor iXON 897E EMCCD camera and Andor iQ 2.4.2 imaging software. The time lapse acquisition mode of the software was used to follow fluorescence changes over time.

### Analysis of Calcium Imaging Data

Images acquired through Andor iQ 2.4.2 on Olympus IX81-ZDC2 were analyzed with ImageJ 1.43m (NIH, Bethesda, MD, USA). Each cell was marked separately and mean fluorescence intensity was calculated in arbitrary units. Arbitrary units of fluorescence for each cell were converted to ΔF/F_0_ values and the highest ΔF/F_0_ values were tabulated; ΔF/F_0_ values for each assay and each genotype were plotted as a box plot or cumulative frequency distribution plot in Origin 8.0 software (Origin Lab, Northampton, MA, USA). To compare data between genotypes or assay conditions Kruskal-Wallis test for variance followed by Wilcoxon *post hoc* test was performed.

### Quantitative PCR

Total RNA was isolated from 5 to 10 dissected CNS from suitably aged *Drosophila* pupae with TRIzol reagent (Invitrogen, Life Technologies, Carlsbad, CA, USA) following manufacturer’s instructions. RNA was dissolved in nuclease free water and quantified using a nanodrop machine (Thermo Scientific, Wilmington, DC, USA) and the integrity was checked on a 1.5% TAE gel. Approximately 200 ng RNA was used for cDNA preparation by Reverse Transcriptase as described in Pathak et al. (2015). Quantitative PCR (qPCR) was performed by ABI 7500 fast machine operated by ABI 7500 software using KAPA^™^ SYBR^®^ FAST qPCR master mix (Kapa Biosystems, Wilmington, MA, USA) for SYBR assay I dTTP (Eurogentec, Belgium). For each genotype, three biological replicates and two technical replicates were included in the experiment. *rp49* was used as internal control. A melt curve was performed after the assay to check for specificity of the reaction. The fold change of gene expression in test genotype relative to control was determined by the comparative DDCt, where DDCt = (Ct_target_ − Ct_p49_)_test_ − (Ct_target_ − Ct_rp49_)_control_.

### Quantitative Western Blots

Pupal CNS lysates were extracted in 50 mM Tris (pH 8), 150 mM NaCl, 1 mM EGTA and 1% (v/v) Triton X-100 with 1 mM PMSF, 0.5 μM E64 Calpain inhibitor and 100 μg/ml leupeptin. Protein extract was boiled for 5 min at 95°C in 125 mM Tris pH 6.8, 5% (v/v) Glycerol, 0.25% (w/v) SDS, 2% (v/v) β-mercaptoethanol, 10% (w/v) Bromophenol blue and resolved in 8% SDS polyacrylamide gel electrophoresis. Proteins were detected with 1:5000 anti-TRP (kind gift from Prof. Raghu Padinjat, NCBS).

### Flight Test

Flies were anesthetized in ice before tethering to a tungsten wire between the head and thorax and allowed to recover for 30 min before recording flight. Flight was stimulated with a puff of air and recorded for 30 s.

## Results

### Pupal Neurons with Mutant IP_3_R Exhibit Increased Spontaneous Ca^2+^ Signals and Reduced Store-Operated Ca^2+^ Entry

**Figure 1:**
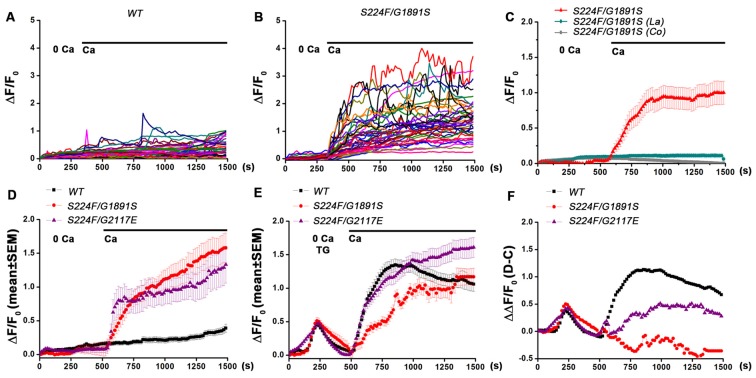
***Drosophila* pupal neurons with mutant IP_3_R exhibit increased spontaneous Ca^2+^ signals and reduced store-operated Ca^2+^ entry (SOCE). (A,B)** Traces showing spontaneous Ca^2+^ signals in individual pupal neurons in absence and presence of extracellular Ca^2+^ in wild type and *itpr* mutant respectively. **(C)** Mean spontaneous Ca^2+^ signals in *itpr* mutant (S224F/G1891S) neurons in presence of La and Co with extracellular Ca^2+^. **(D)** Mean spontaneous Ca^2+^ signals in wild type and *itpr* mutant neurons. **(E)** Mean Ca^2+^ signals in wild type and *itpr* mutant neurons due to store depletion with thapsigargin followed by SOCE. **(F)** Ca^2+^ signals for SOCE after subtracting spontaneous Ca^2+^ influx from Ca^2+^ influx induced by store-Ca^2+^ depletion with thapsigargin (mean data obtained from several separate experiments with same time intervals for recording). *N* > 100 cells.

**Figure 2:**
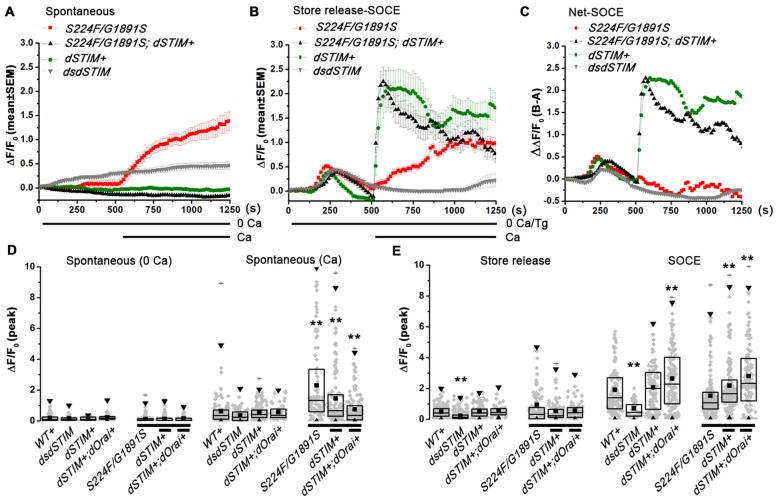
**Aberrant spontaneous Ca^2+^ influx and SOCE in *itpr* mutant pupal neurons can be rescued by overexpression of *dSTIM* and *dOrai*. (A,B)** Traces showing spontaneous Ca^2+^ signals and thapsigargin-Ca^2+^ signals respectively (mean ± SEM) in pupal neurons (*N* > 100). **(C)** Ca^2+^ signals for SOCE after subtracting spontaneous Ca^2+^ influx **(A)** from Ca^2+^ influx induced by store-Ca^2+^ depletion with thapsigargin **(B)**. **(D,E)** Box plot representing peak ΔF/F_0_ with individual values for single neurons, mean, median and 25–75 quartiles for spontaneous Ca^2+^ influx, thapsigargin induced store depletion and SOCE, *N* > 100 cells, *P* > 0.01 compared to wild type or the mutant, Kruskal-Wallis test for variance followed by Wilcoxon *post hoc* test. **P < 0.01.

To explore the role of IP_3_-mediated Ca^2+^ signaling in spontaneous Ca^2+^ influx and SOCE in *Drosophila* pupal neurons, primary neurons from the central nervous systems of 48–50 h pupae were cultured for 16–18 h and Ca^2+^ signals of single neurons were recorded. In presence of 2 mM extracellular CaCl_2_ wild type and *itpr* mutant neurons displayed spontaneous Ca^2+^ signals in culture (Figures 1A,B). The amplitude of signals was significantly higher in neurons with mutant IP_3_Rs (S224F/G1891S and S224F/G2117E; heteroallelic combinations) as compared with wild type (*P* < 0.01, Kruskal-Wallis test of variation followed by Wilcoxon *post hoc* test; Figures 1A,B,D, 2D). The spontaneous Ca^2+^ signals observed in both genotypes were sustained rather than transients. Spontaneous Ca^2+^ signals were not observed in minimal extracellular Ca^2+^ (8–11 nM) and after application of either 50 μM lanthanum chloride (Prakriya and Lewis, 2001) or 2 mM cobalt chloride (Jiang et al., 2005) in presence of 2 mM extracellular CaCl_2_ (Figure 1C), suggesting that the spontaneous Ca^2+^ signals observed were due to a Ca^2+^ influx across the PM and not from intracellular stores. This observation is consistent with published data reporting an absence of spontaneous Ca^2+^ transients in the absence of extracellular Ca^2+^ in cultured *Drosophila* pupal neurons of mushroom body Kenyon cells (Jiang et al., 2005). However, the frequency and amplitude of spontaneous Ca^2+^-signals observed in our study differed from what was reported in mushroom body Kenyon cells (Jiang et al., 2005), possibly due to variations in spontaneous Ca^2+^-signals in specific subtypes of neurons and culture conditions.

*Drosophila* larval neurons cultured from *itpr* mutants (S224F/G1891S; heteroallelic combination) displayed reduced SOCE after passive depletion of intracellular store-Ca^2+^ with 10 μM thapsigargin (Venkiteswaran and Hasan, 2009; Chakraborty and Hasan, 2012a, b; Chakraborty et al., 2016). To understand if SOCE is also compromised in pupal neurons from *itpr* mutants, intracellular store-Ca^2+^ was depleted with 10 μM thapsigargin in minimal extracellular Ca^2+^ (8–11 nM) followed by measurement of SOCE by replenishing 2 mM extracellular CaCl_2_. Because *itpr* mutant pupal neurons displayed higher spontaneous Ca^2+^ influx as compared to wild type in presence of extracellular Ca^2+^, SOCE was determined by subtracting spontaneous Ca^2+^ influx from Ca^2+^ influx induced by store-Ca^2+^ depletion with thapsigargin (Figure 1E). As in larval neurons, SOCE was significantly (*P* < 0.01, Kruskal-Wallis test of variation followed by Wilcoxon *post hoc* test) reduced in *itpr* mutant pupal neurons (Figure 1F).

### Over-Expression of *dSTIM/dOrai* in *itpr* Mutant Neurons Restores Normal Spontaneous Ca^2+^ Influx and Store-Operated Ca^2+^ Entry

To understand if spontaneous Ca^2+^ influx in pupal neurons is affected by SOCE, spontaneous Ca^2+^ influx and SOCE was measured in pupal neurons over-expressing *dSTIM* and *dOrai* in presence or absence of mutant IP_3_Rs (Figure 2). Higher spontaneous Ca^2+^ influx was significantly reduced by over-expression of *dSTIM* (Figures 2A,D) and *dOrai* (Figure 2D) in *itpr* mutant neurons, suggesting that their higher spontaneous Ca^2+^ influx is a compensatory increase for reduced SOCE. This idea was tested further, by measuring spontaneous Ca^2+^ influx and SOCE in pupal neurons with knock down of *dSTIM*. Knock down of *dSTIM (dsdSTIM)* reduced SOCE in pupal neurons (Figures 2C,D); however, it did not result in significant up regulation of spontaneous Ca^2+^ influx (Figures 2A,D). Thus the higher spontaneous Ca^2+^ influx in *itpr* mutant neurons appears to be an adaptive effect of recurrent loss of IP_3_R function rather than a response to loss of SOCE. Loss of SOCE in *itpr* mutant larval neurons can be restored by over-expression of *dSTIM* and *dOrai* (Agrawal et al., 2010). Over-expressing *dSTIM* and *dOrai* in wild type and *itpr* mutant pupal neurons also increased SOCE after store-Ca^2+^ depletion with thapsigargin (Figures 2B,C,E). As published in larval neurons (Chakraborty et al., 2016), over-expression of *dSTIM* and *dOrai* in *itpr* mutant neurons did not alter store-Ca^2+^ depletion in pupal neurons. However, knock down of *dSTIM* attenuated intracellular store Ca^2+^ release presumably due to reduced store [Ca^2+^] (Figures 2B,C,E). These data suggest that loss of SOCE, in absence of a compensatory increase in spontaneous Ca^2+^ influx as seen in neurons with *dSTIM* knock-down (Figure 2A), leads to reduced Ca^2+^ in intracellular stores. This reiterates that increased spontaneous Ca^2+^ entry in *itpr* mutant neurons helps refill ER store in the absence of SOCE.

### IP_3_R Function/SOCE Regulate Expression of PM Ca^2+^ Channels in *Drosophila* Pupal Neurons

**Figure 3:**
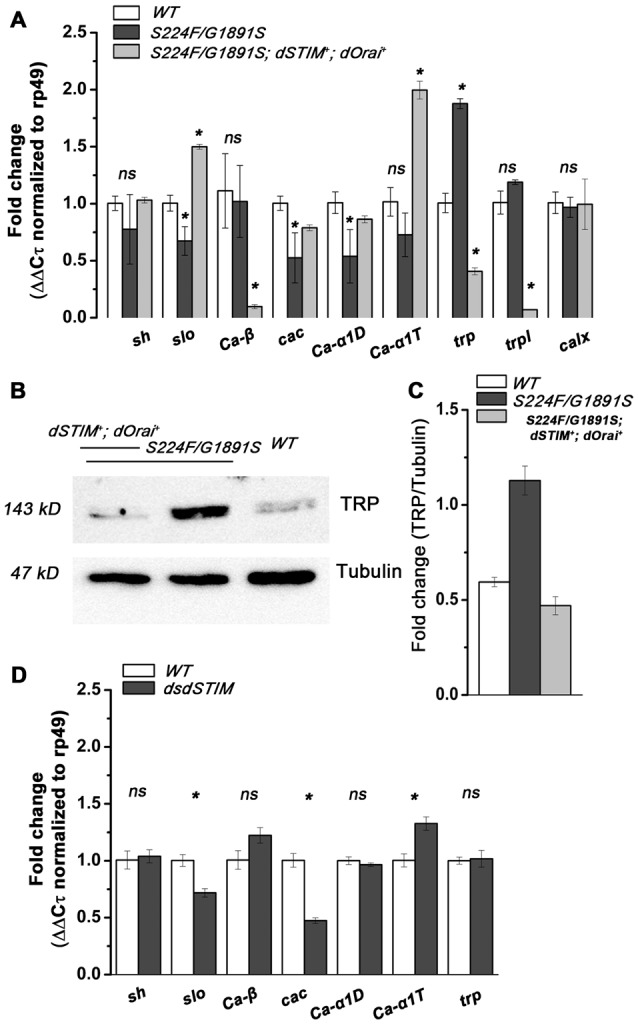
**IP_3_R function/SOCE regulates expression of plasma membrane (PM) Ca^2+^ channels in *Drosophila* pupal neurons. (A)** Fold change (ΔΔ*C*τ normalized to rp49 mRNA, mean ± SEM) of mRNA levels for *Slowpoke* and *Shaker*, subunits of VGCCs (*Ca-β, cacophony/cac*, *Ca-α1D* and *Ca-αT*), TRP channels (*trp, trpl*) and NCX (*calx*) in extracts derived from 48–50 h pupal central nervous system in the respective genotypes, *N* = 3, Student’s *t*-test. **(B)** Representative western blot showing expression of TRP in the respected genotypes. **(C)** Fold change of TRP protein expression (mean ± SEM) in the respective genotypes, *N* = 3, Student’s *t*-test. **(D)** Fold change (ΔΔ*C*τ normalized to rp49 mRNA, mean ± SEM) of mRNA levels for *Slowpoke* and *Shaker*, subunits of VGCCs (*Ca-β, cacophony/cac*, *Ca-α1D* and *Ca-αT*) and TRP in the respective genotypes, *N* = 3, Student’s *t*-test. *P < 0.05.

To identify PM Ca^2+^ channels that contribute to higher spontaneous Ca^2+^ influx in *Drosophila* *itpr* mutant pupal neurons, we performed quantitative PCR analysis of mRNA encoding subunits of VGCCs (*Ca-β, cacophony/cac*, *Ca-α1D* and *Ca-α1T*), TRP channels (*trp, trpl*) and NCX (*calx*) in extracts derived from 48 h to 50 h pupal central nervous systems. The choice of candidates investigated was based on existing literature (Gu et al., 2009; Iniguez et al., 2013; Kanamori et al., 2013). The time window selected was based on previous data on the requirement of IP_3_R function and SOCE for the development of the *Drosophila* flight circuit (Banerjee et al., 2004; Richhariya et al., 2017) and on the onset of spontaneous Ca^2+^ transients in *Drosophila* pupal neurons (Jiang et al., 2005). Spontaneous Ca^2+^ transients in distinct subsets of *Drosophila* pupal neurons (*in vivo* and in 2 day old cultures) have been attributed to PLTX-II sensitive P/Q-type VGCC, *cac* (Jiang et al., 2005; Gu et al., 2009) or to T-type VGCC, *Ca-αT* (Iniguez et al., 2013). An increased activity of voltage-activated transient K^+^ current *I*_A_ was observed in *cac* mutant neurons, associated with an increased expression of S*haker*, gene for *I*_A_, and a corresponding reduction in the expression of *Slowpoke*, gene for [*I*K_(*Ca*)_] (Peng and Wu, 2007). Hence expression levels of K^+^ channel *Slowpoke* and *Shaker* were also analyzed as reporters for VGCC activity. Surprisingly, a significant reduction in *cacophony/cac*, *Ca-α1D* and *Ca-αT* mRNA levels was observed in *itpr* mutant neurons with a corresponding reduction in *Slowpoke mRNA*. Expression of *Ca-β* and *Shaker*, however, was not significantly altered (*P* < 0.05, Student’s *t*-test; Figure 3A). These data suggest that higher spontaneous Ca^2+^ influx in *itpr* mutant neurons is very likely not mediated by VGCCs. On the contrary, the normal expression of VGCCs correlates with normal IP_3_R function.

The TRPC class of PM Ca^2+^ channels are responsible for Ca^2+^ spike activity in developing *Xenopus* spinal cord, where Shh signaling induces synchronous Ca^2+^ spikes and IP_3_ transients at the primary cilium (Belgacem and Borodinsky, 2011). NCX (Na^+^/Ca^2+^ exchanger) expression is indirectly modulated by SOCE, through ERK1/2, in neuronal PC12 (Sirabella et al., 2012) and rat parotid acinar cells (Soltoff and Lannon, 2013). Therefore, the levels for mRNAs encoding two *Drosophila* TRPC proteins; TRP and TRPL (Thebault et al., 2005) and a *Drosophila* NCX homolog, CalX (Wu et al., 2011) were tested. A significant increase (*P* < 0.05, Student’s *t*-test) in mRNA levels of *trp* was observed in *itpr* mutant neurons, whereas expression levels of *trpl* and *calx* remained unchanged (Figure 3A). Over-expression of TRP in the pupal nervous system of *itpr* mutants was confirmed by measuring expression of TRP protein directly (Figures 3B,C).

Interestingly, rescue of SOCE by over-expression of *dSTIM* and *dOrai* in *itpr* mutant neurons reverted the change in mRNA levels of *cacophony/cac*, *Ca-α1D*, *Slowpoke* and *trp* (*P* < 0.05, Student’s *t*-test; Figure 3A). Moreover, expression of *Ca-α1T* and *trpl* (*P* < 0.05, Student’s *t*-test; Figure 3A) was also altered significantly suggesting that raising SOCE in *itpr* mutant neurons, by overexpression of *dSTIM* and *dOrai*, altered gene expression of VGCCs and TRPs. To understand if expression levels of *cacophony/cac*, *Ca-α1D*, *Slowpoke* and *trp* were directly sensitive to SOCE, mRNA levels were analyzed from pupal neurons with down regulation of *dSTIM* (Figure 3D). A significant (*P* < 0.05, Student’s *t-test*) down regulation of *cac* and *Slowpoke* was observed in *dSTIM* knocked down neurons whereas expression levels of *Shaker, Ca-β, Ca-α1D* and *trp*, remained unchanged. These data suggest that the expression of *cac* and *slo* are sensitive to SOCE. The reduced expression of *Slowpoke* possibly reports reduced activity of the P/Q-type VGCC encoded by *cac*, as reported earlier for *cac* mutant neurons (Peng and Wu, 2007). However, it is also possible that *Slowpoke* expression in this case is directly regulated by SOCE rather than through reduced activity of VGCCs. The absence of TRP up-regulation, as well as normal spontaneous Ca^2+^ influx in neurons with *dSTIM* knock-down supports higher expression of TRP in *itpr* mutant neurons as a likely cause for higher spontaneous Ca^2+^ influx. Over-expression of TRP thus appears to be sensitive to recurrent loss of IP_3_R function rather than loss of SOCE in *Drosophila* pupal neurons. These data support a central role for the IP_3_R in maintaining calcium homeostasis in *Drosophila* pupal neurons.

### Knock-Down of VGCCs in Global or Subset of Neurons Does Not Affect *Drosophila* Flight

**Figure 4:**
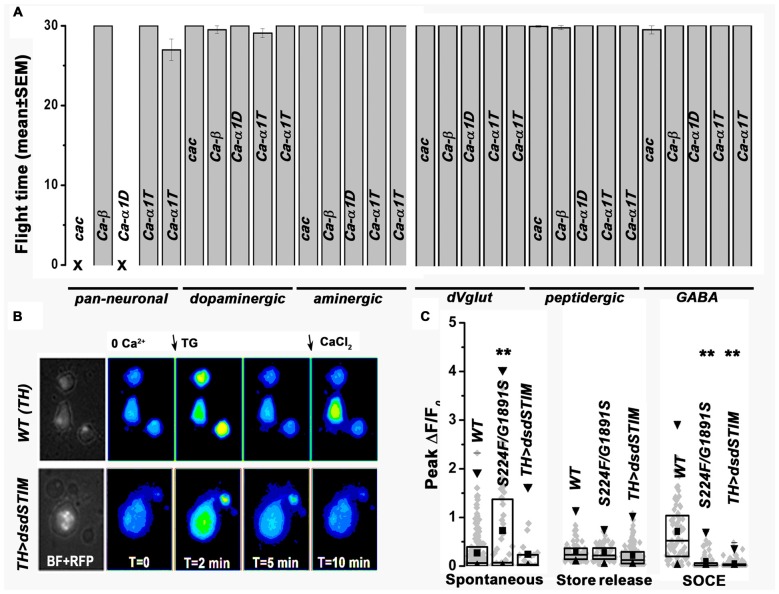
**Neuronal knock-down of VGCCs does not affect *Drosophila* flight. (A)** Flight time (mean ± SEM) with knockdown of VGCCs respectively in the mentioned neurons, *cac* (104186), *Ca-β* (102188), *Ca-α1D* (51491) and *Ca-α1T* (48008, 31961), *N* > 30 individual flies, *P* > 0.05, Kruskal-Wallis test for variance followed by Wilcoxon *post hoc* test. **(B)** Representative images showing store-depletion by thapsigargin and SOCE in TH positive pupal neurons in the indicated genotypes. **(C)** Box plot representing peak ΔF/F_0_ with individual values for single neurons, mean, median and 25–75 quartiles for spontaneous Ca^2+^ influx, thapsigargin induced store depletion and SOCE respectively, *N* > 50 cells, *P* > 0.01, Kruskal-Wallis test for variance followed by Wilcoxon *post hoc* test. **P < 0.01.

Previous results have demonstrated that knock down of either *itpr* or *dSTIM* in *Drosophila* neurons during pupal development leads to flight deficits in adults (Agrawal et al., 2013; Richhariya et al., 2017). Reduced expression of *cac* in *itpr* mutant neurons and in neurons with knock-down of *dSTIM* suggested that *cac* down regulation may be associated with the observed deficits in *Drosophila* flight. To test this hypothesis, we measured flight times in animals with knock-down of *cac* and other subunits of VGCCs in either all or subsets of *Drosophila* neurons (Figure 4A). Pan-neuronal knock-down of *cac* (104186) and *Ca-α1D* (51491) resulted in 100% lethality in late stage 3rd instar larvae and thus these animals could not be tested for flight. Pan-neuronal knock-down of *Ca-β* (102188) and *Ca-α1T* (48008, 31961) did not affect either viability or flight. We also tested flight after knock-down of these VGCC subunits in specific neuronal subsets. The RNAi lines used were based on their efficacy as described in published literature (Gu et al., 2009; Iniguez et al., 2013; Kanamori et al., 2013). Knockdowns were performed in dopaminergic, aminergic, glutamatergic, peptidergic and GABA-ergic neuronal subsets, which include neuronal classes in which IP_3_R and SOCE function has been previously implicated as required for adult flight (Banerjee et al., 2004; Venkiteswaran and Hasan, 2009; Agrawal and Hasan, 2015; Pathak et al., 2015). Flight times were tested up to 30 s after air-puff stimulation of tethered flight and appeared normal in all the knock down genotypes tested. Thus, the observed down regulation of VGCCs (Figures 3A,D) correlates with reduced SOCE (Figure 2E) but does not affect flight circuit function (Figure 4A). Knock-down of TRP in *itpr* mutant neurons and its effect on spontaneous Ca^2+^ influx and flight in *Drosophila* could not be assessed as the recombinant flies with TRP RNAi and *itpr* mutants didn’t survive.

### Spontaneous Ca^2+^ Influx in Pupal Dopaminergic Neurons Is Associated with Loss of Flight

Recent work investigating the role of intracellular calcium signaling in flight has shown that SOCE is required in dopaminergic neuronal subsets for transcriptional maturation of the *Drosophila* flight circuit during pupal development (Pathak et al., 2015). Therefore, we tested spontaneous Ca^2+^ influx specifically in dopaminergic neurons of *itpr* mutant and *dSTIM* knockdown pupae (Figures 4B,C). As observed in all pan-neuronal populations (Figures 1, 2), spontaneous Ca^2+^ influx in pupal dopaminergic neurons from a mutant IP_3_R (S224/G1891S) was significantly higher, whereas SOCE was lower as compared to wild-type dopaminergic neurons (Figure 4C). The IP_3_R is required in *Drosophila* neurons during pupal development for flight (Agrawal et al., 2013). Spontaneous Ca^2+^ signals in *Drosophila* neurons also originate during late stage pupal development (Jiang et al., 2005). Our data support a model where loss of flight in *itpr* mutant flies may be an outcome of the up regulation of spontaneous Ca^2+^ influx during pupal development possibly resulting in an imbalance of excitatory and inhibitory neurotransmitters in the developing flight circuit.

## Discussions

*Drosophila* pupal neurons mutant for IP_3_R exhibit greater spontaneous Ca^2+^ influx as compared with wild type (Figures 1A,B,D) and lack SOCE even after equivalent depletion of intracellular store upon passive depletion with thapsigargin (Figures 1E,F). Our results suggest that recurrent loss of IP_3_R function and SOCE in *Drosophila* pupal neurons triggers compensatory spontaneous Ca^2+^ entry to maintain intracellular Ca^2+^ homeostasis. Restoration of SOCE by over-expression of *dSTIM* and *dOrai* restores spontaneous Ca^2+^ influx to wild type levels in *itpr* mutant neurons and also rescues flight (Figures 2A–E; Richhariya et al., 2017, respectively). However, pan-neuronal knock down of *dSTIM* with a GAL4 strain that expresses in post-mitotic neurons (Lin and Goodman, 1994) didn’t result in higher spontaneous Ca^2+^ entry (Figure 2A), indicating that loss of SOCE alone does not trigger higher spontaneous Ca^2+^ influx. Previous results from the lab demonstrate that over-expression of *dSTIM* and *dOrai* in *Drosophila* larval neurons could rescue Ca^2+^ release through IP_3_R (Agrawal et al., 2010). Thus, restoration of spontaneous Ca^2+^ influx in *Drosophila* pupal neurons could be an outcome of restored IP_3_R function by over-expression of *dSTIM* and *dOrai*. The cellular mechanisms underlying these observations need further elucidation. Our data suggest that loss of IP_3_R function and loss of SOCE, with the consequent reduction in intracellular store Ca^2+^ in *dSTIM* knocked down neurons, may influence different aspects of cell function.

We have established that expression of VGCCs and TRP are modulated by IP_3_R function and can be restored by raising SOCE in *Drosophila* pupal neurons (Figures 3A–C). Down regulation of SOCE through knock down of *dSTIM* altered expression of VGCCs, however did not alter expression of TRP (Figure 3D). The functional significance of reduced VGCCs expression in *itpr* mutant neurons (Figure 3A) is unclear because knock-down of VGCCs in *Drosophila* neurons (global and subsets) didn’t result in flight deficits (Figure 4A). Our results suggest that higher spontaneous Ca^2+^ influx in *itpr* mutant neurons is likely due to up regulation of PM Ca^2+^ channel TRP, and this may contribute to the observed flight deficits. The mechanism that triggers higher expression as well as spontaneous activation of TRP needs further exploration.

Involvement of dopaminergic neurons in *Drosophila* flight has been described earlier where reduced SOCE in pupal dopaminergic neurons resulted in reduced expression of Tyrosine Hydroxylase, required for synthesis of dopamine (Pathak et al., 2015). As observed in pan-neuronal population (Figures 1, 2), spontaneous Ca^2+^ influx in pupal dopaminergic neurons from a mutant IP_3_R (S224/G1891S; Figure 4C) was significantly higher, whereas SOCE was lower as compared to wild-type dopaminergic neurons (Figure 4C). Knock down of *dSTIM (dsdSTIM) in* dopaminergic neurons reduced SOCE (Figures 4B,C); however, it did not result in significant up regulation of spontaneous Ca^2+^ influx (Figure 4C). Thus, higher spontaneous Ca^2+^ influx in dopaminergic neurons could be one of multiple contributing factors to the flight deficits of *Drosophila* *itpr* mutants. It is known that spontaneous Ca^2+^ influx has a direct role in neurotransmitter specification and motor co-ordination in vertebrate neurons (Spitzer et al., 2005). Possibly, the higher spontaneous Ca^2+^ influx in *itpr* mutant neurons is an adaptive effect of dual loss of IP_3_R function and the accompanying loss of SOCE. Clearly, loss of SOCE alone (by knockdown of *dSTIM*) does not induce higher spontaneous influx. Compromised IP_3_R function and SOCE in *Drosophila* neurons leads to defects in flight motor coordination (Banerjee et al., 2004; Venkiteswaran and Hasan, 2009). However, the effect of IP_3_R-mediated Ca^2+^ release on neuronal properties during neural circuit development has not been explored. Here we show that *Drosophila* pupal neurons mutant for IP_3_R display higher spontaneous Ca^2+^ influx, which can be restored by over expression of *dSTIM* and *dOrai*. Direct measurement of electrophysiological properties of adult flight circuit neurons is required to understand the effect of increase in spontaneous Ca^2+^ influx during pupal development on these neurons. Loss of IP_3_R function in the vertebrate nervous system leads to defects in motor coordination and spino-cerebellar ataxia (SCA); a deletion mutation of *ITPR1* in human causes SCA15/16 (van de Leemput et al., 2007; Novak et al., 2010), and the mouse knock out (KO) for *Itpr1* displays ataxia (Matsumoto et al., 1996). The connection between IP_3_R function, spontaneous Ca^2+^ signals and SOCE provides a novel perspective to address fundamental questions in neurodegenerative diseases and offers new targets for subsequent development of therapeutics.

## Author Contributions

SC conceived the project, designed and performed experiments, contributed reagents, analyzed data and interpreted results, prepared figures and drafted the manuscript. GH supervised SC, contributed reagents and critically evaluated the data and the manuscript.

## Funding

The work was supported by core funding from National Centre for Biological Sciences (NCBS) to GH. SC was supported by senior research fellowship from Council of Scientific and Industrial Research (CSIR), India and a bridging postdoctoral fellowship from NCBS.

## Conflict of Interest Statement

The authors declare that the research was conducted in the absence of any commercial or financial relationships that could be construed as a potential conflict of interest.

## Abbreviations

IP_3_R: inositol 1,4,5-trisphosphate receptor
SOCE: store-operated calcium entry
STIM: stromal interaction molecule
TRP: transient receptor potential
VGCCs: voltage gated calcium channels.
